# Problématique de la prise en charge des fièvres hémorragiques: expérience de la maladie à virus Ebola dans la province du Nord Kivu et Ituri (République Démocratique du Congo) et importance du diagnostic précoce

**DOI:** 10.11604/pamj.2021.39.240.21195

**Published:** 2021-08-13

**Authors:** Criss Koba Mjumbe, Isabelle Kasongo Omba, Ghyslain Ngongo Lambo, Francis Mbuyi Kolela, Chadrack Kabeya Diyoka, Oscar Luboya Nuymbi

**Affiliations:** 1 Département de Santé Publique, Faculté de Médecine de Lubumbashi, Lubumbashi, République Démocratique du Congo,; 2 Département de Médecine Interne, Polyclinique Saint Joseph/GCM, Lubumbashi, République Démocratique du Congo

**Keywords:** Prise en charge, Ebola, Nord Kivu, diagnostic précoce, Management, Ebola, North Kivu, early diagnosis

## Abstract

La maladie à virus Ebola est une maladie grave, souvent mortelle, dont le taux de létalité peut atteindre 90%. L´objectif à court terme de cette lettre aux auditeurs est de faire connaitre les signes du virus Ebola à la population et de favoriser le diagnostic précoce dans notre milieu. Nous avons appliqué une observation des cas de l´épidémie Ebola dans notre milieu. Il n´est pas toujours possible d´identifier rapidement les patients présentant une maladie à virus Ebola. Pour cette raison, il est important que les agents de santé appliquent les précautions d´usage à tous les patients, quel que soit le diagnostic, dans toute pratique professionnelle et à tout moment. Avec l´appui du gouvernement congolais et des plusieurs autres organismes, le gouvernent congolais devrait lancer un programme de sensibilisation des masses et de vaccination contre le virus Ebola.

## Abstract (en)

Ebola virus disease is a serious, often fatal disease whose case-fatality rate can be 90%. Our short-term goal is to create awareness on Ebola virus signs and to encourage early diagnosis in our milieu. We observed Ebola virus disease cases in our community. It is not always possible to quickly identify patients with Ebola virus disease. For this reason, it is essential that healthcare providers use standard precautions for all patient care, regardless of diagnosis, in any professional practice and at all times. The government of the Democratic Republic of the Congo supported by several other organizations should implement mass awareness and vaccine programs against Ebola virus.

## Aux éditeurs du Pan African Medical Journal

La maladie à virus Ebola est une maladie grave, souvent mortelle, dont le taux de létalité peut atteindre 90% [1]. Comme son nom l´indique, elle est due au virus Ebola, qui appartient à la famille des filovirus. Cependant, même dans les pays développés où des différentes recherches sont effectuées en matière de la prise en charge, Ebola reste encore un grand défi de la médecine actuellement, car il est généralement greffé d´une lourde mortalité. En Afrique de l´Ouest, l´épidémie qui a sévi en 2014, affichait une létalité de 39,5% avec 11 323 morts sur 28 646 cas recensés [2]. Lors de la dernière flambée en Guinée suite à une souche Ebola, 90% des patients sont décédés de la maladie [3]. Peu d´études épidémiologiques sont rapportées en Afrique en général, la plupart des pays ne possédant pas de registres nationaux d´enregistrement annuel. Quelques études hospitalières donnent néanmoins une idée des statistiques. Le virus Ebola appartient au genre d´Ebolavirus de la famille des Filoviridae (filovirus), à laquelle appartient également le virus Marburg. Tous sont des virus à l´apparence filamenteuse caractéristique. On en distingue six types espèces virales [4]. À l´intérieur du genre Ebolavirus: *Ebolavirus Bundibugyo, Ebolavirus foret de Taï, Ebolavirus Reston, Ebolavirus Soudan, Ebolavirus Zaire et Ebolavirus Bombali* [5].

Il existe donc une réelle opportunité de survie si le diagnostic est posé précocement et le traitement entrepris à temps. L´expérience rapportée dans le Nord Kivu de la RD Congo traités conjointement au centre de traitement Ebola (CTE) de Butembo. Le virus Ebola est un virus relativement difficile à diagnostiquer. La transmission interhumaine du virus Ebola est avant tout liée au contact direct ou indirect avec du sang et des liquides biologiques. Il n´est pas toujours possible d´identifier rapidement les patients présentant une maladie à virus Ebola car les symptômes initiaux peuvent manquer de spécificité. Pour cette raison, il est important que les agents de santé appliquent les précautions d´usage à tous les patients, quel que soit le diagnostic, dans toute pratique professionnelle et à tout moment. Les signes d´appels sont parfois une hémorragie importante et une défaillance des organes qui peuvent entrainer la mort. Des symptômes que la population et le personnel soignant devraient identifier et reconnaitre dès le départ sont: douleurs dans certaines zones tel que l´abdomen, articulations, muscles ou poitrine; dans le corps entier: chair de poule, déshydratation, fatigue, fièvre, inconfort physique, perte d´appétit ou transpiration ; gastro-intestinaux : diarrhée, nausées, vomissement ou vomissement sanguinolents ; et enfin les symptômes courants confusion mentale, crachats de sang en toussant, mal de gorge, maux de tête, tache rouge sur la peau ou yeux rouge. La confirmation du diagnostic est faite après examen de laboratoire, mais avant il reste un cas suspect. Une fois diagnostiqué au stade précoce, plus de 80% des cas sont correctement pris en charge avec les mesures de sécurité pour éviter toute contamination et de fois on assiste à une guérison [6]. Le pronostic est réservé au stade tardif.

**Figure 1:**
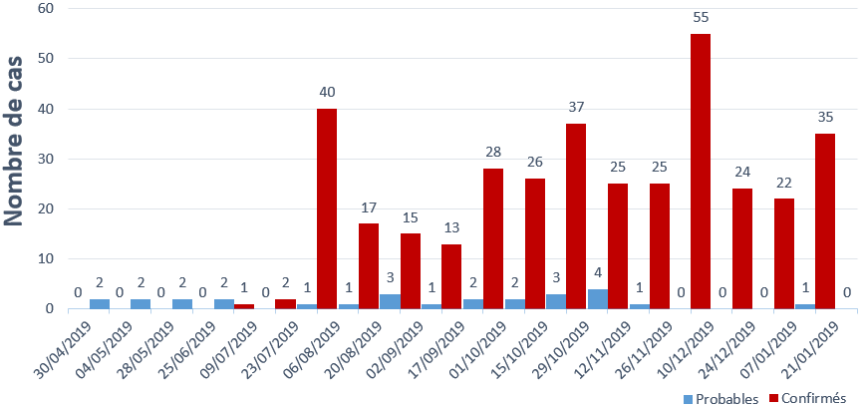
cas et décès confirmés et probable de la maladie à virus Ebola par date de début de signes, du 1^er^ mai 2018 au 27 janvier 2019, Province du Nord Kivu et Ituri

Selon le Tableau 1, incluant 716 cas (682 confirmés et 34 probables) dont 459 décès, soit une létalité de près de 64,1% parmi les cas confirmés et probables. La létalité parmi les cas confirmés était de 75,3%. Sur les 430 cas observés chez les femmes, 47,4% (204) sont des femmes en âge de procréation (15 à 49 ans d´âge). Les enfants représentent un nombre disproportionné de cas par rapport à l´épidémie précédente. Trente-huit pour cent (n=167, 38,8%) des cas sont âgés de moins de 18 ans, et 13,7% (n=59) sont des jeunes enfants de moins de 5 ans. Les agents de santé n´ont pas été épargnés par le virus, 65 cas soit 9,1% de cas. La Figure 1 rapporte la chronologie des cas confirmés et probables et des décès dus à la maladie à virus Ebola par date d'apparition des signes, du 21 janvier 2019 au 24 décembre 2019 dans les provinces du Nord-Kivu et de l'Ituri. La difficulté liée au diagnostic du virus Ebola est commune à plusieurs pays africains frappés par ce virus. Une étude indiquait qu´en Guinée, au Liberia et en Sierra Leone, l´épidémie avait déjà dépassé plus de 15 000 morts [6]. Les résultats étaient similaires dans une étude au Nigeria qui montre une telle incidence [7]. Il est un fait que la République Démocratique Congo est un pays à ressources limitées. Néanmoins, les mesures pouvant améliorer la riposte et la prise en charge et infléchir la mortalité liée à Ebola sont très accessibles. Il existe des atouts qui peuvent déjà être exploités. À Kinshasa il existe une unité de virologie à l´Institut National de Recherche Biomédicale (IRNB) qui se penche sur la question. Elle est parrainée par le Gouvernement congolais et certains bailleurs de fond. Elle bénéficie d´une pluridisciplinarité avec différents services. Le défi pour Ebola dans le contexte de notre milieu, reste le rôle de la sensibilisation et de l´éducation des masses. C´est probablement ce sur quoi doivent encore œuvrer les prestataires des soins et la politique nationale de santé. En l´occurrence, dans le cas du virus Ebola, un transfert immédiat des malades présentant les signes d´appel au centre de référence est indispensable. La systématisation des examens virologiques permettrait de cibler précocement la population à risque.

**Tableau 1:** fréquence de patients atteints du virus Ebola

Paramètres		Effectif	%
Confirmé		682	95,3
Probable		34	4,7
Taux de létalité chez les cas confirmés et probables		459	64,1
Sexe	Masculin	286	39,9
	Féminin	430	60,1
**Total**		**716**	**100**
